# Dual-ended readout TOF-DOI PET detectors based on 3.2 mm and 1.6 mm pitch LYSO arrays

**DOI:** 10.1186/s40658-025-00759-y

**Published:** 2025-05-27

**Authors:** Haibo Wang, Jiahao Xie, Jinyi Qi, Simon R. Cherry, Junwei Du

**Affiliations:** Department of Biomedical Engineering, University of California at Davis, Davis, CA 95616 USA

**Keywords:** Positron emission tomography (PET), Time-of-flight (TOF), Depth-of-interaction (DOI), Coincidence timing resolution (CTR), Dual-ended readout

## Abstract

**Background:**

The image quality of positron emission tomography (PET) can be significantly enhanced by using time-of-flight (TOF) and depth-of-interaction (DOI) information. PET detectors are pivotal in determining the TOF and DOI capabilities of PET scanners.

**Methods:**

This study developed and evaluated TOF-DOI PET detectors based on the dual-ended readout method and lutetium-yttrium oxyorthosilicate (LYSO) arrays with two different pitches and reflector configurations. Specifically, the performance of detectors based on three types of LYSO arrays with 20 mm thickness, 8 × 8 arrays with a 3.2 mm pitch, 16 × 16 arrays with a 1.6 mm pitch and normal reflectors, and 16 × 16 arrays with a 1.6 mm pitch and partial short reflectors, were assessed. Hamamatsu S14161-3050-08 silicon photomultiplier arrays were used as the photodetectors, and PETsys TOFPET2 was used as the readout electronics.

**Results:**

The flood histograms showed that all crystals in the three types of LYSO arrays were clearly resolved. The detectors based on the 8 × 8 LYSO arrays provided a coincidence timing resolution (CTR) of 207 ± 5 ps and a DOI resolution of 3.9 ± 0.6 mm. The detectors based on the 16 × 16 LYSO arrays with normal reflectors provided a CTR of 218 ± 7 ps and a DOI resolution of 2.6 ± 0.2 mm. In comparison, the detector based on the 16 × 16 LYSO arrays with partial short reflectors provided a CTR of 228 ± 11 ps and a DOI resolution of 2.9 ± 0.3 mm, and superior crystal resolvability compared to the detectors based on the 16 × 16 LYSO arrays with normal reflectors.

**Conclusion:**

These detectors are promising candidates for developing whole-body and brain PET scanners, offering effective sensitivity and uniform spatial resolution improvements across the field-of-view.

## Background

Positron emission tomography (PET) is a molecular imaging technique enabling *in vivo* visualization of biological processes [1]. The image quality of PET scans, often measured by the signal-to-noise ratio (SNR), is influenced by counting statistics, which are affected by the injected radiotracer dose, scan time, and system sensitivity [2]. Optimizing these factors is crucial for enhancing image quality and minimizing patient radiation exposure [3, 4].

**Fig. 1:**
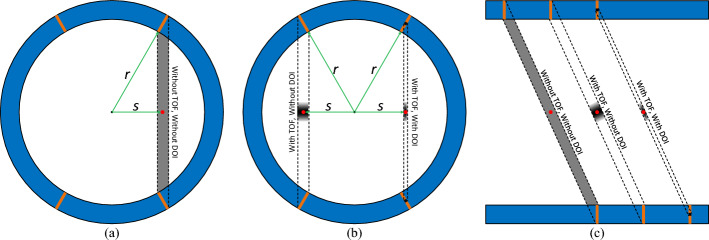
Compared to without using TOF and DOI information, the estimation of the positron annihilation position in **a**,**b** the radial direction and **c** the axial direction can be greatly improved by using TOF and DOI information, which improves the image quality. The red dots show the positron annihilation position, while the gray areas show the estimated positron annihilation position

Recent advancements in whole-body PET instrumentation technology have aimed at maximizing sensitivity through the use of thicker crystals to improve detector detection efficiency [5, 6], extended axial field-of-view (FOV) [6–8], and improved time-of-flight (TOF) capabilities [6, 9, 10]. The increment in detector detection efficiency follows an exponential drop-off relative to crystal thickness, which means that there is a point at which additional thickness provides diminishing returns on detection efficiency. Meanwhile, the use of thicker crystals increases the cost of the PET system. Currently, most TOF-PET scanners utilize ~ 20 mm thick lutetium oxyorthosilicate (LSO) or lutetium-yttrium oxyorthosilicate (LYSO) crystals to balance cost and detection efficiency [6, 7, 9]. Extending the axial length of scanners, as seen in the total-body EXPLORER PET scanners and the Siemens Biograph Vision Quadra PET scanners [6, 7], can significantly increase the sensitivity, though at a proportionate cost increase. The TOF technique enhances effective sensitivity by precisely determining the positron annihilation position along the line-of-response (LOR) through the measured time difference between annihilation photons arriving at the detectors (Fig. 1) [11–13]. The accuracy of this method is directly linked to the coincidence timing resolution (CTR) of the PET system. Using TOF information can provide a gain increase for the system's sensitivity characterized by

1 $${Sensitivity}_{gain}\propto \frac{D}{{c\delta }_{t}},$$

where *D* is the diameter of the imaged subject, $c$ is the speed of light, and ${\delta }_{t}$ is the CTR. Thus, better CTR can lead to better contrast recovery, lesion detectability, and quantitative accuracy [14], and the benefits of using TOF information have been well-established [11, 15–18].

Additionally, PET image quality is compromised by parallax errors when the depth-of-interaction (DOI) in the thick crystal is unknown [19, 20]. This parallax error degrades the radial spatial resolution at locations in the scanner that are radially off-center (Fig. 1), especially for scanners with smaller diameters, such as dedicated brain PET scanners [20–23], and the axial spatial resolution of long axial FOV scanners. Various detector design techniques have been proposed to provide DOI information, including dual-ended readout [24–26], multi-layer crystals [27–30], monolithic or semi-monolithic crystals [31, 32], and complex-shaped reflectors [30, 33]. Among these, the dual-ended readout method is notable for its ability to achieve continuous and accurate DOI resolutions by using the ratio of signals detected by photodetectors coupled to both ends of the crystal or crystal arrays [24, 34]. Moreover, leveraging DOI information can enhance the CTR by correcting the annihilation photons' interaction position [35–37]. Hence, the development of PET detectors with excellent CTR and DOI resolution is important and significant for next-generation high-performance PET scanners.

This work introduces three TOF-DOI PET detectors based on LYSO arrays with two different pitches (3.2 mm and 1.6 mm) and two reflector arrangements (normal reflectors and partial short reflectors). These detectors, developed for next-generation whole-body PET and dedicated PET scanners, such as brain PET scanners, leveraged the dual-ended readout method to provide superior CTR and DOI resolution. The performance of the detectors was comprehensively evaluated through flood histogram, energy resolution, CTR, and DOI resolution measurements. Additionally, the CTRs with and without using inter-crystal scatter (ICS) events, and with and without performing time-walk and timing-shift corrections, were compared.

## Dual-ended readout PET detectors

### LYSO arrays and SiPM arrays

**Fig. 2:**
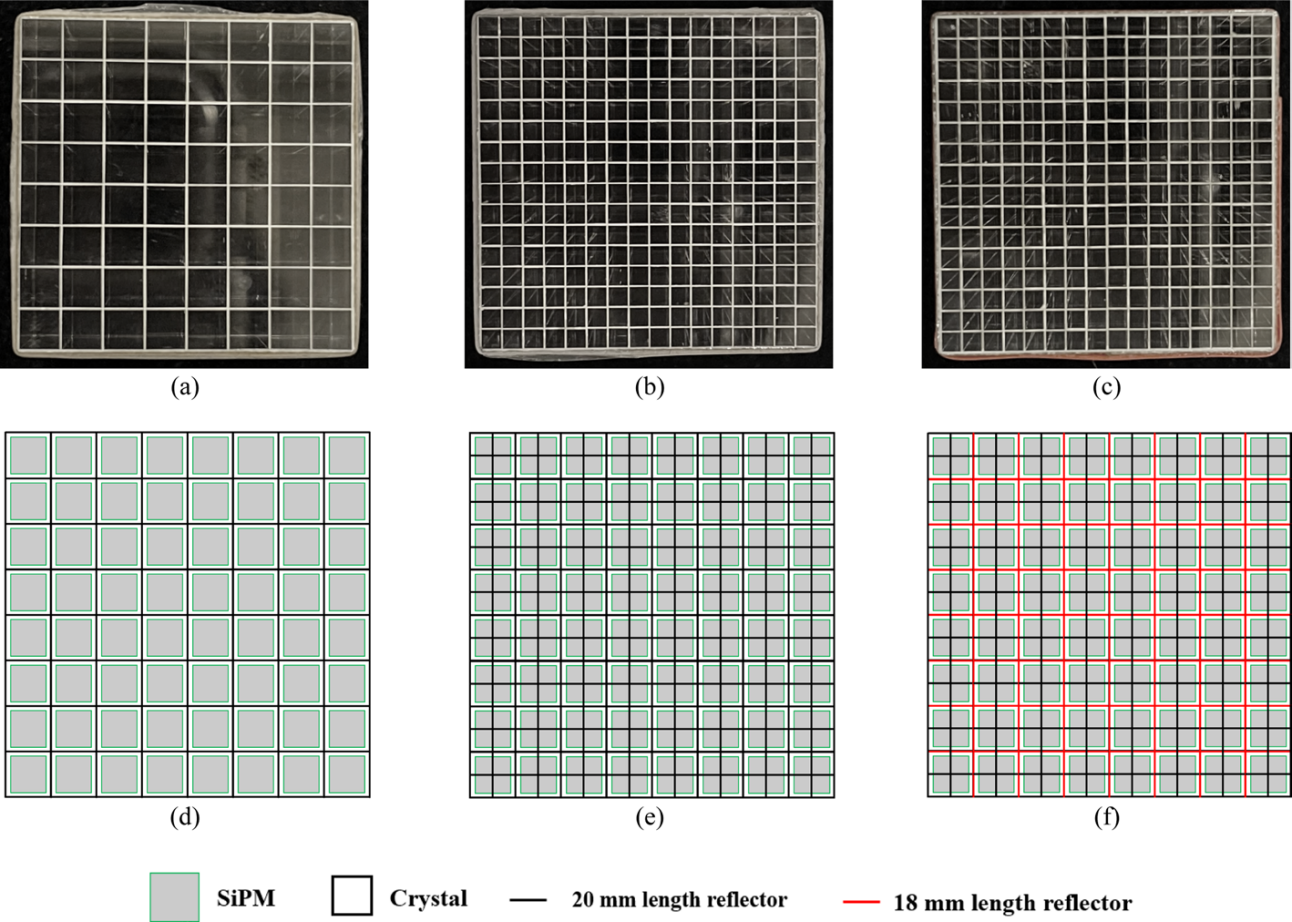
Photographs of **a** an 8 × 8 array of 3.1 × 3.1 × 20 mm^3^ LYSO, **b** a 16 × 16 array of 1.5 × 1.5 × 20 mm^3^ LYSO with normal reflectors, and **c** a 16 × 16 array of 1.5 × 1.5 × 20 mm^3^ LYSO with partial short reflectors. Schematics of the coupling configuration between the SiPM arrays and **d** the 8 × 8 LYSO array, **e** the 16 × 16 array with normal reflectors, and **f** the 16 × 16 array with partial short reflectors. The black and red lines indicate reflectors with 20 mm and 18 mm lengths, respectively. The gray square areas indicate the SiPMs, smaller than the 3.0 × 3.0 mm^2^ active area of the SiPMs to show the coupling configurations clearly

**Fig. 3:**
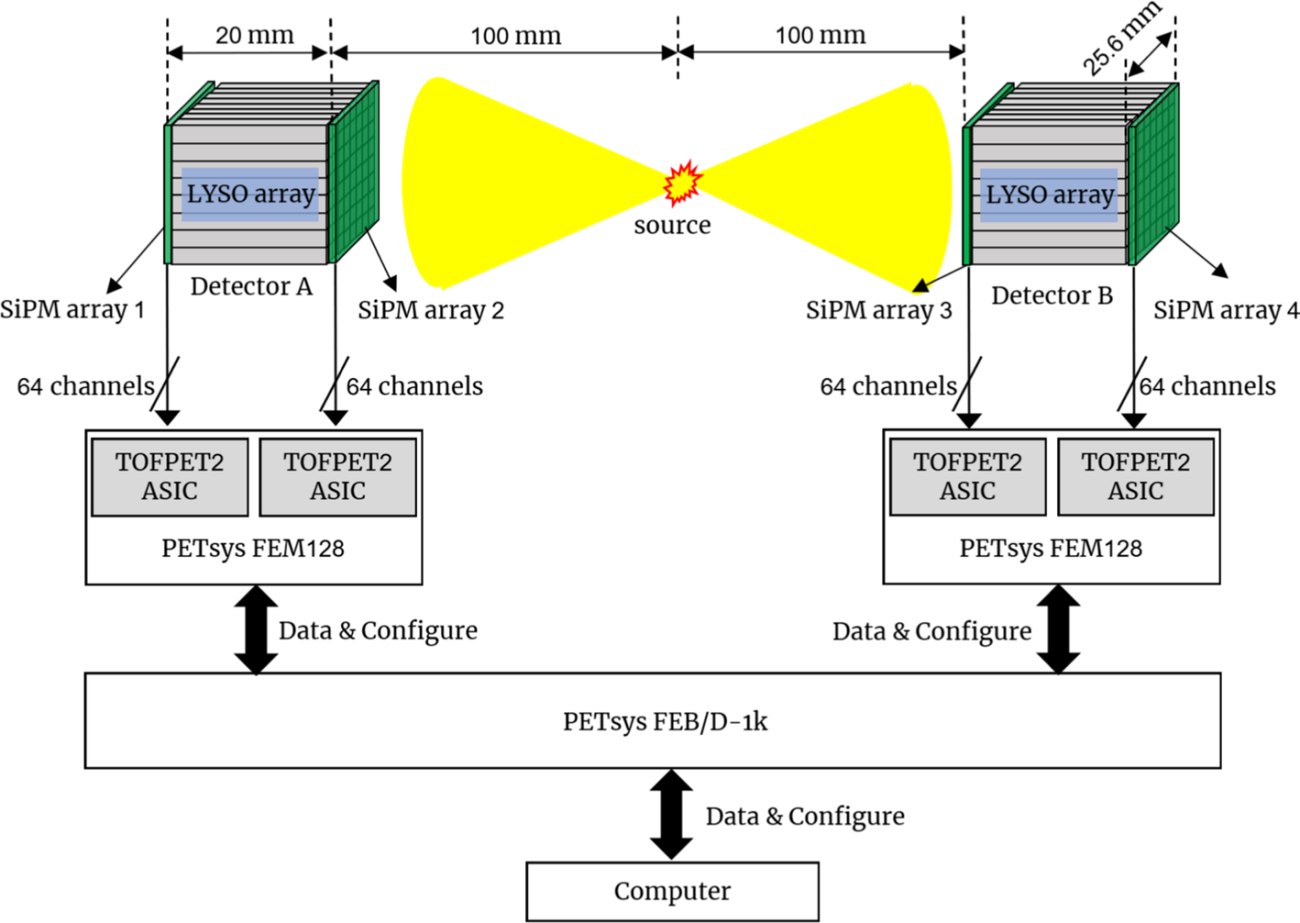
Experimental setup for flood histogram, energy resolution, and CTR measurements. Distances and object sizes are not to scale

Figure 2 shows the three types of LYSO arrays and their coupling configurations with the silicon photomultiplier (SiPM) arrays employed in this study. All LYSO elements were polished on all six surfaces, and all LYSO arrays were fabricated using the same fabrication techniques. Barium sulfate (BaSO_4_) with a 0.1 mm thickness was used as the reflector between LYSO elements. Hamamatsu S14161-3050-08 SiPM arrays (Hamamatsu Photonics K.K.) were used as the photodetectors and were coupled to both ends of the LYSO arrays to assemble dual-ended readout detectors (Fig. 3). Optical grease BC-630 (Luxium Solutions, LLC) was applied between the LYSO arrays and SiPM arrays in all assembled detectors.

The details of the three types of LYSO arrays are as follows:

- The first type of LYSO array, shown in Fig. 2a, was an 8 × 8 array of 3.1 × 3.1 × 20 mm^3^ LYSO crystals with a 3.2 mm pitch. Due to the LYSO arrays and the SiPM arrays having the same 3.2 mm pitch, each LYSO element was coupled to two SiPM elements, one for each end of the LYSO element, to achieve the best CTR (Fig. 2d). Detectors based on this type of LYSO array were designed for TOF-DOI whole-body PET scanners, as the crystal size was similar to those used in state-of-the-art whole-body PET scanners [6, 9]. This type of LYSO array was referred to as the "8 × 8 LYSO array" throughout the paper.
- The second type of LYSO array, shown in Fig. 2b, was a 16 × 16 array of 1.5 × 1.5 × 20 mm^3^ LYSO crystals with a 1.6 mm pitch. Hence, for a dual-ended readout detector based on this type of LYSO array, four crystal elements were coupled to two SiPM elements (one at each end) (Fig. 2e). Detectors based on this type of LYSO array were designed for next-generation whole-body PET scanners that can push the spatial resolution of whole-body PET scanners to the next level. These detectors can also be used for dedicated PET scanners, such as brain PET scanners. Compared to detectors based on the first type of LYSO array, the photodetectors and readout electronics were the same, but the smaller size crystal will give a better spatial resolution. This type of LYSO array was referred to as the "16 × 16 LYSO array with normal reflectors" throughout the paper.
- The third type of LYSO array, shown in Fig. 2c, was also a 16 × 16 array of 1.5 × 1.5 × 20 mm^3^ LYSO crystals with a 1.6 mm pitch. However, to better resolve the LYSO elements, 18 mm length reflectors, 1 mm shorter than the crystals at both ends, were used between alternating LYSO elements, aligned with the gaps of the SiPMs, as indicated by the red lines in Fig. 2f. The 1 mm reserved length at both ends was filled with optical glue, forming an internal light guide to enhance light sharing between the SiPMs, which is expected to improve crystal identification [38]. This LYSO array was referred to as the "16 × 16 LYSO array with partial short reflectors" throughout the paper.

### Readout electronics

The PETsys TOFPET2 (PETsys Electronics S.A.) evaluation kit was used to process the SiPM signals, enabling individual readout for each SiPM signal. Briefly, the output from the two Hamamatsu S14161-3050-08 SiPM arrays of one dual-ended readout detector was connected to one PETsys FEM128 module containing two TOFPET2 application-specific integrated circuits (ASICs). The output of the FEM128 module was then sent to a PETsys FEB/D-1k board, which can support up to 8 FEM128 modules (Fig. 3). Each TOFPET2 ASIC has 64 channels and can independently process the 64 SiPM signals from one Hamamatsu S14161-3050-08 SiPM array. Each ASIC channel includes independent amplifiers, discriminators, time-to-digital converters (30 ps time bin), and charge-to-digital converters. More details about the PETsys TOFPET2 ASIC can be found in [39, 40].

## Experimental methods

For each type of LYSO array, two identical detectors were constructed, and the same four Hamamatsu SiPM arrays were used as the photodetectors to eliminate any variations in SiPM performance affecting the results. During all measurements, the detector under test and the PETsys FEM128 modules were located in a light-tight and temperature-controlled enclosure. The temperature inside the enclosure was maintained at 20 ± 0.5 °C, as measured by the temperature sensor on the PETsys FEM128 modules. A 925 kBq (25 µCi) ^22^Na point source with an active diameter of 0.25 mm was used to irradiate the LYSO arrays. The SiPM arrays were biased at 41.5 V, and a lower threshold of 5 DAC bin was used for timing pick-off.

### Flood histogram, energy resolution, and CTR measurements

The flood histogram, energy resolution, and CTR were measured using two identical detectors in a face-to-face configuration, as shown in Fig. 3. The distance between the two detectors was 200 mm, and the source was located at the center between them. The same event dataset was used to determine the flood histogram, energy resolution, and CTR.

#### Flood histogram

The annihilation photon interaction positions, *x* and *y*, within the LYSO array were calculated using the center-of-gravity (COG) method and signals detected by all triggered SiPMs using the formulae:

2 $$\left\{\begin{array}{l}x=\frac{1}{2}\left(\frac{\sum_{i=1}^{64}{s}_{1,i}^{n}{x}_{1,i}}{\sum_{i=1}^{64}{s}_{1,i}^{n}}+\frac{\sum_{i=1}^{64}{s}_{2,i}^{n}{x}_{2,i}}{\sum_{i=1}^{64}{s}_{2,i}^{n}}\right)\\ y=\frac{1}{2}\left(\frac{\sum_{i=1}^{64}{s}_{1,i}^{n}{y}_{1,i}}{\sum_{i=1}^{64}{s}_{1,i}^{n}}+\frac{\sum_{i=1}^{64}{s}_{2,i}^{n}{y}_{2,i}}{\sum_{i=1}^{64}{s}_{2,i}^{n}}\right)\end{array},\right.$$

where *i* (*i* = 1…64) represented the order of SiPM elements in one SiPM array, ${s}_{1,i}$ and ${s}_{2,i}$ represented the signals detected by SiPM *i* of the two SiPM arrays coupled to both ends of the LYSO array, and ${x}_{1,i}$, ${y}_{1,i}$, ${x}_{2,i}$ and ${y}_{2,i}$ represented the positions of SiPM *i* in the two SiPM arrays coupled to both ends of the LYSO array. The power *n* was used to improve the crystal identification ability [41–43]. In our study, flood histograms obtained using *n* with three different values, 1, 1.5, and 2, were compared. When *n* = 1, Eq. (2) represented the conventional COG method.

#### Energy resolution

For each event, the deposited energy *E* was calculated as the sum of signals detected by all fired SiPMs from the two SiPM arrays of the dual-ended readout detector. To calculate the energy resolution for each crystal, events were assigned to each crystal using crystal look-up tables, and then energy spectra were generated for each crystal. The energy resolution was quantified as the ratio of the full width at half maximum (FWHM) to the peak position of the 511 keV photopeak derived from a Gaussian fit. For each detector, the average energy resolution across all 64 or 256 crystals was used to characterize the energy resolution of the detector.

#### Coincidence timing resolution

The timing difference ($\Delta t$) of two annihilation photons from a positron decay was calculated using the following formulae [44, 45]:

3 $$\Delta t={t}_{A}-{t}_{B},$$

4 $${t}_{A}=({t}_{1}+{t}_{2})/2,$$

5 $${t}_{B}=({t}_{3}+{t}_{4})/2,$$

where ${t}_{A}$ and ${t}_{B}$ denoted the timing information of detectors A and B, respectively, and ${t}_{i}$ (*i* = 1…4) represented the threshold crossing time (timestamps) from the four SiPM arrays of the two detectors in coincidence (Fig. 3). When more than one SiPM in a SiPM array was triggered and multiple timestamps were generated, the timestamp associated with the highest detected signal was used.

Due to the use of leading-edge discriminators (LEDs) by the PETsys TOFPET2 for timing pick-off, the threshold crossing time ${t}_{i}$ suffered from time-walk. Additionally, due to the DOI effect and the offsets between different channels of the PETsys TOFPET2, the threshold crossing time ${t}_{i}$ also suffered from timing-shift. Therefore, the timing difference $\Delta t$ was affected by both time-walk and timing-shift, which needed to be corrected to improve the CTR. The detailed procedures for correcting time-walk and timing-shift were described in our previous studies [46].

**Fig. 4:**
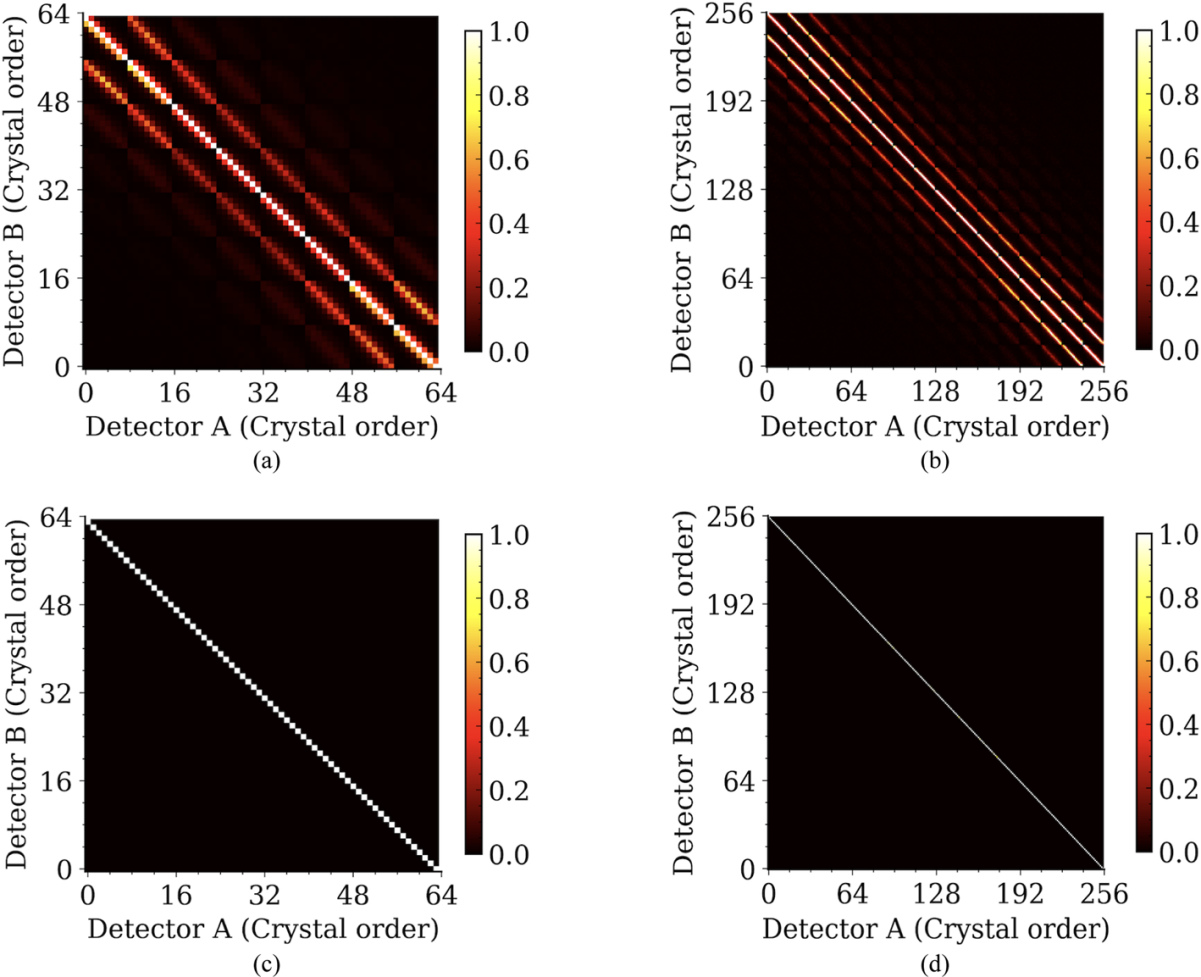
Normalized counts for each crystal pair of detectors based on (**a**) the 8 × 8 LYSO arrays and (**b**) the 16 × 16 LYSO arrays with normal reflectors. The crystal pairs along the diagonal with sufficient events were used to calculate the CTR for detectors based on (**c**) the 8 × 8 LYSO arrays and (**d**) the 16 × 16 LYSO arrays with normal reflectors. The results for detectors based on the 16 × 16 LYSO arrays with partial short reflectors were similar to those shown in (**b**) and (**d**)

Since a point source was used in our experiment, only some crystal pairs detected sufficient coincidence events for calculating the CTR, as shown in Fig. 4, which displays the event counts for all possible crystal pairs. The diagonal patterns observed in Figs. 4c, d correspond to crystal pairs aligned along the LOR passing through the point source and with sufficient events. Hence, in our timing analysis, only crystal pairs ${C}_{i}^{A}{C}_{i}^{B}$ (*i* = 1…64 for detectors based on the 8 × 8 LYSO arrays and *i* = 1…256 for detectors based on the 16 × 16 LYSO arrays) located along the diagonal lines shown in Fig. 4 were used to analyze the CTR [25].

CTR, defined as the FWHM of the Gaussian fit to the timing spectrum, was calculated for each selected crystal pair (Figs. 4c, d) [25]. The CTRs with and without performing time-walk and timing-shift corrections, as well as with and without using ICS events, were also compared.

#### ICS event identification

**Fig. 5:**
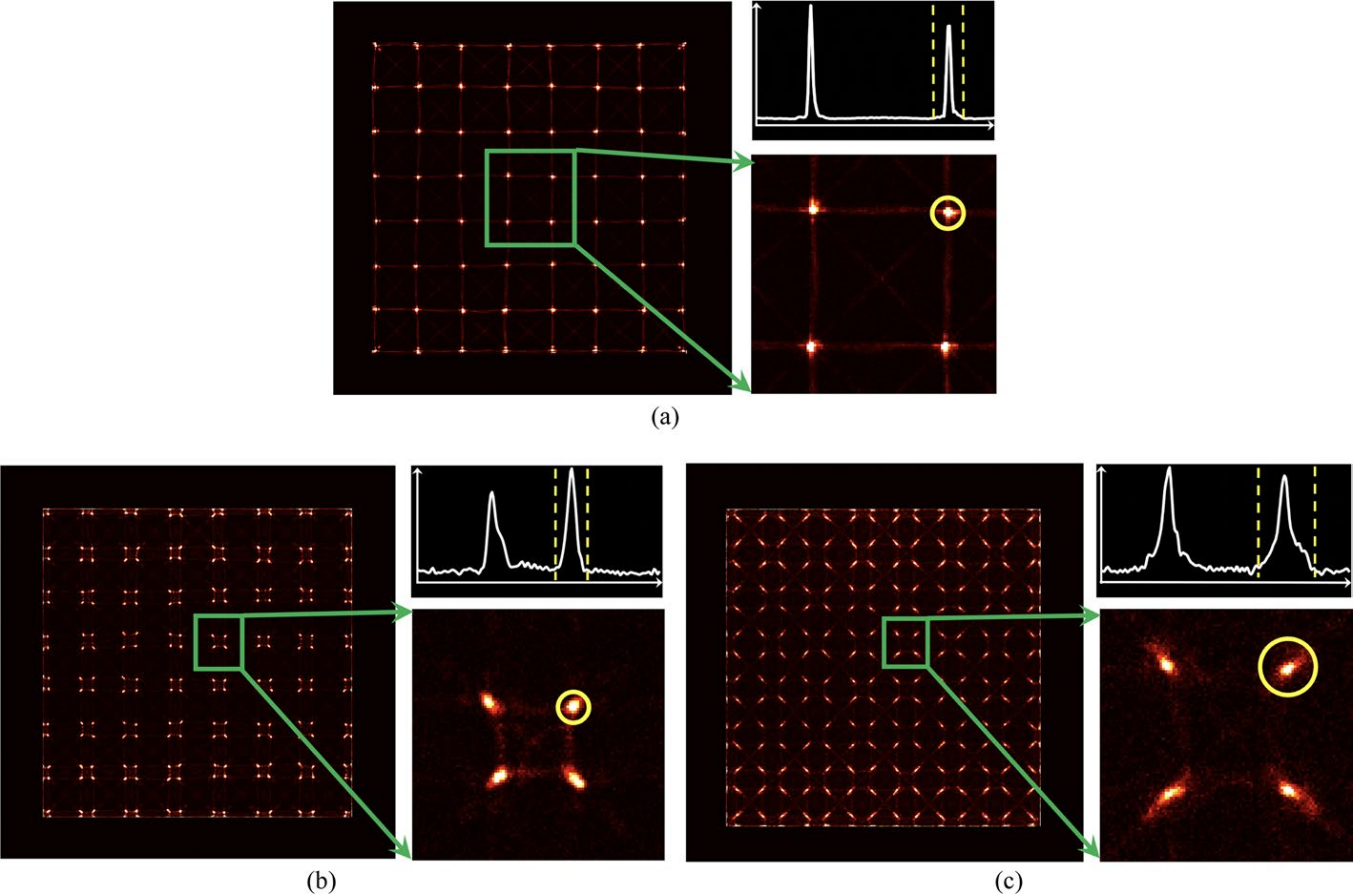
The method based on masks, shown as yellow circles around the center of the crystal spots, was used to exclude ICS events. If an event was not inside the masks, it was treated as an ICS event. For the detectors based on (**a**) the 8 × 8 LYSO array, (**b**) the 16 × 16 LYSO array with normal reflectors, and (**c**) the 16 × 16 LYSO array with partial short reflectors, the diameters of the masks were 20, 20, and 30 pixels, respectively

ICS events undergo Compton scattering and deposit their energies into multiple LYSO elements of the detector [47]. Consequently, ICS events result in cross-box patterns across the crystal spots in the flood histogram, as shown in Fig. 5. To exclude ICS events, two different approaches are widely used:

- Based on the number of triggered SiPMs: If one annihilation photon triggers more than two SiPMs in a dual-ended readout PET detector, this event can be categorized as an ICS event. However, this method could only be used for detectors based on the 3.2 mm pitch LYSO array. For detectors based on the 1.6 mm pitch LYSO arrays, since four LYSO elements were coupled to two SiPMs, ICS among those crystals coupled to the same SiPM could not be identified. Additionally, due to scintillation light cross-talk among crystals, even if an event deposits its energy into one crystal, more than two SiPMs could be triggered. Hence, this method overestimated the ICS ratio for 3.2 mm pitch LYSO arrays.
- Based on the position of the event in the flood histogram: As shown in Fig. 5, because ICS events deposit their energies into multiple LYSO elements, their COGs fall between the crystal spots in the flood histogram. Thus, a mask could be used to reject ICS events [47, 48]. This method can be applied to any type of detector, and it was selected for our studies. However, the drawback of this method is that the ratio of ICS events is highly dependent on the size of the mask. If the crystal spot does not have a circular shape, it is difficult to draw the mask. To avoid discarding non-ICS events, a large enough circular mask is usually used, as was the case in our studies. Hence, the ICS event ratio was underestimated using this method in our studies.

We used circular masks with diameters of 20, 20, and 30 pixels applied to each crystal spot to remove the ICS events for the detectors based on the 8 × 8 LYSO arrays (Fig. 5a), the 16 × 16 LYSO arrays with normal reflectors (Fig. 5b), and the 16 × 16 LYSO arrays with partial short reflectors (Fig. 5c), respectively. The size of the flood histograms obtained using the three types of detectors was 1024 × 1024 pixels. Since the flood histograms of the detectors based on the 16 × 16 LYSO arrays with partial short reflectors showed oval crystal spots with tails, larger circular masks were selected. Drawing oval masks based on individual crystal spots is challenging because the shapes of crystal spots appear different. Using this circular mask-based method, 48.4%, 65.3%, and 58.5% of coincidence events were excluded from the detectors based on the 8 × 8 LYSO arrays, the 16 × 16 LYSO arrays with normal reflectors, and the 16 × 16 LYSO arrays with partial short reflectors, respectively. We again highlight that the ratio of ICS events was underestimated in our studies, especially for the 16 × 16 LYSO arrays with partial short reflectors, to avoid excluding non-ICS events from the datasets.

### DOI resolution measurement

**Fig. 6:**
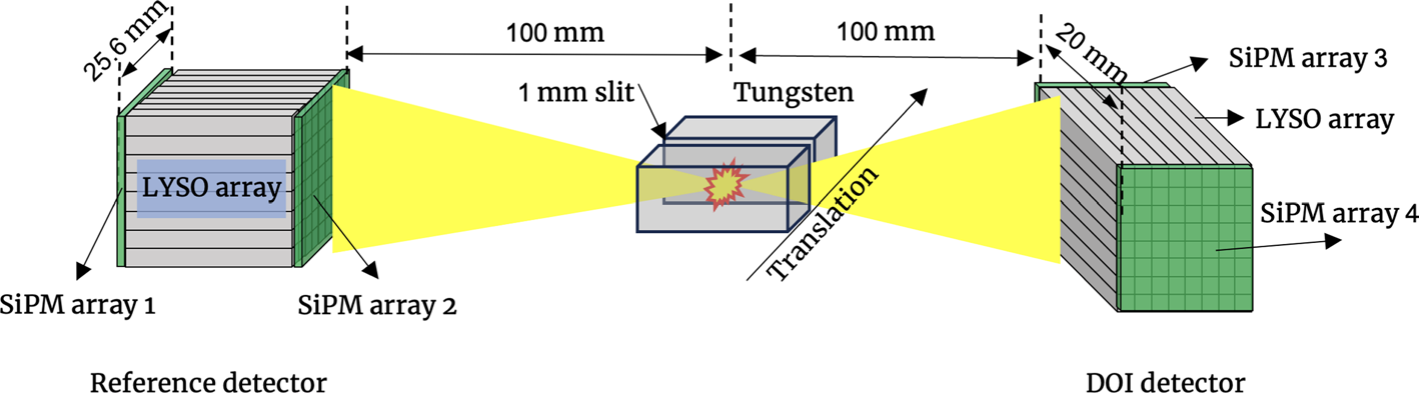
Experimental setup for DOI resolution measurements. Distances and object sizes are not to scale

DOI resolution measurements were performed using one dual-ended readout detector irradiated from one side by rotating this detector by 90°, while the other detector served as a reference detector, as shown in Fig. 6. To irradiate the LYSO arrays at different known depths, the ^22^Na point source was mounted on a linear stage, and two tungsten blocks with a size of 32 × 32 × 64 mm^3^ were positioned on either side of the source. The distance between the two tungsten blocks was 1 mm.

The DOI resolution was obtained at nine depths, ranging from 2 to 18 mm in 2 mm steps. A 400–650 keV energy window was applied to each crystal to select events. The DOI information of the events was calculated based on the detected energies of the two SiPM arrays coupled to both ends of the LYSO array using the following formula:

6 $$DOI\; ratio=a\frac{{E}_{1}-{E}_{2}}{{E}_{1}{ + E}_{2}}+b,$$

where ${E}_{1}$ and ${E}_{2}$ were the two energies detected by the two SiPM arrays coupled to the two ends of the LYSO array, and parameters *a* and *b* were fit parameters used to model the relationship between the DOI ratio and the two detected energies [25].

**Fig. 7:**
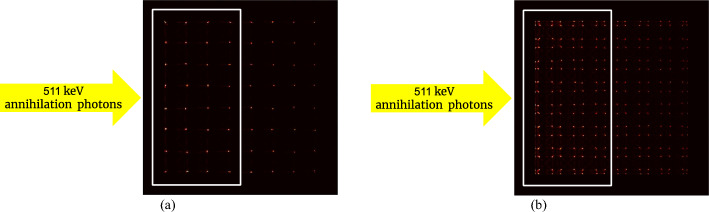
Flood histograms obtained from the DOI measurement: **a** 8 × 4 crystals (white square region) from the detectors based on the 8 × 8 LYSO arrays and (**b**) 16 × 8 crystals (white square region) from the detectors based on the 16 × 16 LYSO array were selected for the DOI resolution analysis

Because the DOI detector was irradiated from one side, only crystals close to the source could detect a sufficient number of events. Therefore, 8 × 4 crystals from the detector based on the 8 × 8 LYSO array (Fig. 7a) and 16 × 8 crystals from the detectors based on the 16 × 16 LYSO arrays (Fig. 7b), as shown in the white square region of Fig. 7, were selected to measure the DOI resolution of the corresponding detector.

## Results

### Flood histogram

**Fig. 8:**
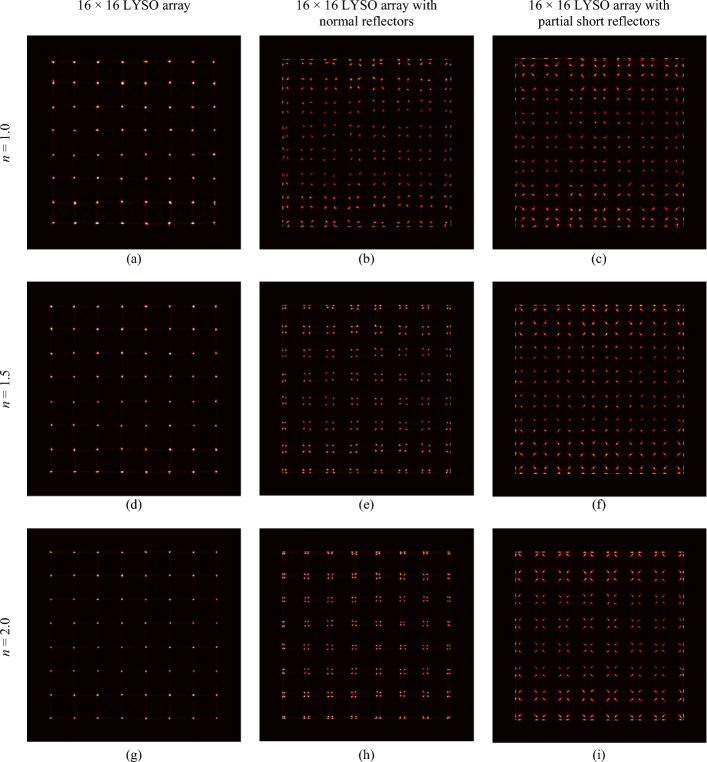
Flood histograms of detectors as a function of weighting factor *n*: (**a**, **d**, **g**) the 8 × 8 LYSO array; (**b**, **e**, **h**) the 16 × 16 LYSO array with normal reflectors; and (**c**, **f**, **i**) the 16 × 16 LYSO array with partial short reflectors

Figure 8 shows the flood histograms of one detector based on the three types of LYSO arrays obtained using weighting factors *n* of 1.0, 1.5, and 2. For the other detector of each pair, the flood histograms were similar to those shown in Fig. 8.

In the detector based on the 8 × 8 LYSO array, each crystal was coupled to two SiPMs, one at each end. Consequently, the crystal spots were evenly distributed in the flood histogram (Figs. 8a, d, g), and no noticeable change in the positions of the crystal spots was observed. However, the crystal spots became smaller when *n* increased.

For the detectors based on the 16 × 16 LYSO arrays, due to four crystals being coupled to one SiPM at each end and the limited scintillation light sharing among SiPMs, the crystal spots were grouped into 2 × 2 clusters in the flood histograms (Figs. 8b, c, e, f, h, i). The distance between the crystal spots in the same 2 × 2 clusters also decreased when the value of *n* increased. This was because the weighting of the SiPM that detected more scintillation light increased with the increasing weighting factor in the COG position estimation algorithm (Eq. (2)). Compared to the 16 × 16 LYSO array with normal reflectors (Figs. 8b, e, h), better flood histograms were obtained using the 16 × 16 LYSO array with partial short reflectors (Figs. 8c, f, i), as these crystals without reflectors between them created an internal light guide that improved scintillation light sharing among SiPMs. Among the three tested weighting factors, the weighting factor *n* with a value of 1.5 provided the overall best visual flood histogram, especially for the edge crystals.

#### 511 keV photopeak position and energy resolution

**Fig. 9:**
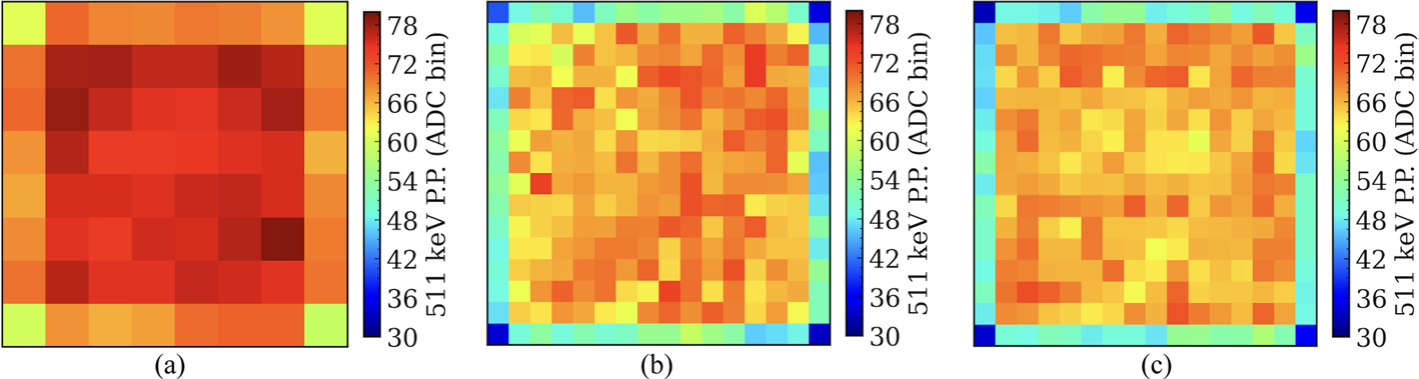
The 511 keV photopeak position (P.P.) for each crystal of detectors based on (**a**) the 8 × 8 LYSO array, (**b**) the 16 × 16 LYSO array with normal reflectors, and (**c**) the 16 × 16 LYSO array with partial short reflectors

**Fig. 10:**
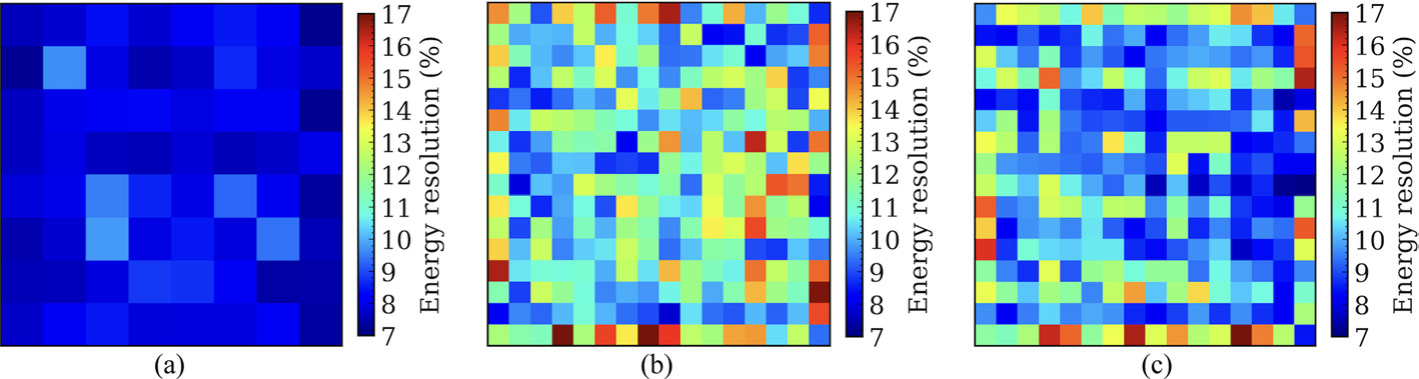
The energy resolution for each crystal of detectors based on (**a**) the 8 × 8 LYSO array, (**b**) the 16 × 16 LYSO array with normal reflectors, and (**c**) the 16 × 16 LYSO array with partial short reflectors

Figures 9 and 10 show the 511 keV photopeak positions and the energy resolutions for each crystal for the three types of LYSO arrays, respectively. The average 511 keV photopeak positions and the average energy resolutions across all crystals for the three types of LYSO arrays are listed in Table 1. No saturation correction was applied, as it is almost impossible to perform this correction for dual-ended readout detectors [49]. The detector based on the 8 × 8 LYSO array provided a higher photopeak position and a better energy resolution than the detectors based on the 16 × 16 LYSO arrays, as the aspect ratio of the LYSO elements was lower (6.5 for 8 × 8 LYSO array vs. 13.3 for 16 × 16 LYSO array). The lower aspect ratio minimized variations in the amount of scintillation light reaching the SiPMs and reduced the chance of scintillation light loss. There was no significant difference both in the average photopeak positions and the average energy resolutions obtained from the two detectors based on the two types of 16 × 16 LYSO arrays.

**Table 1:** The average 511 keV photopeak positions (P.P.) and the average energy resolutions of detectors based on the three types of LYSO arrays

	8 × 8 LYSO array with a 3.2 mm pitch	16 × 16 LYSO array with a 1.6 mm pitch	
		Normal reflector	Partial short reflector
511 keV P.P. (ADC bin)	72.3 ± 4.8	63.1 ± 8.0	62.6 ± 8.1
Energy resolution (%)	8.0 ± 0.6	11.4 ± 2.0	10.5 ± 2.0

For all detectors, the photopeak positions of the edge crystals were lower than those of the center crystals due to some scintillation light escaping from the lateral surface of the LYSO arrays and the cross-section of the LYSO arrays being slightly larger than the active area of the SiPM arrays (Fig. 2), resulting in scintillation light loss.

### Coincidence timing resolution

**Fig. 11:**
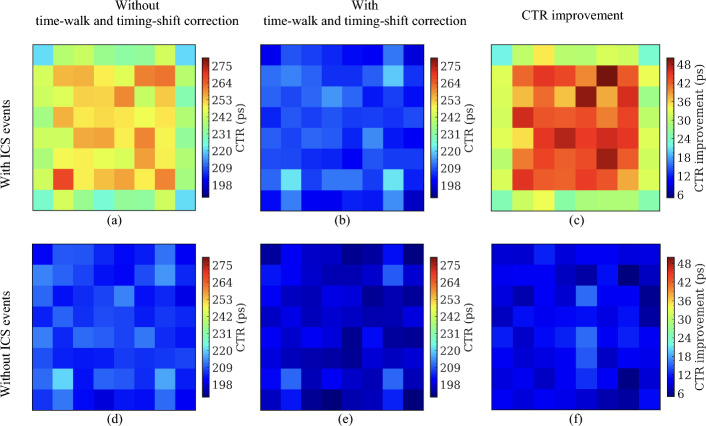
CTR for each of the selected 64 crystal pairs from detectors based on the 8 × 8 LYSO arrays obtained (**a**, **d**) without and (**b**, **e**) with time-walk and timing-shift correction, and (**c**, **f**) the CTR improvement. CTR obtained (**a**, **b**) with ICS events was worse than CTR (**d**, **e**) without ICS events. A 400–650 keV energy window was applied to each crystal to select events

**Fig. 12:**
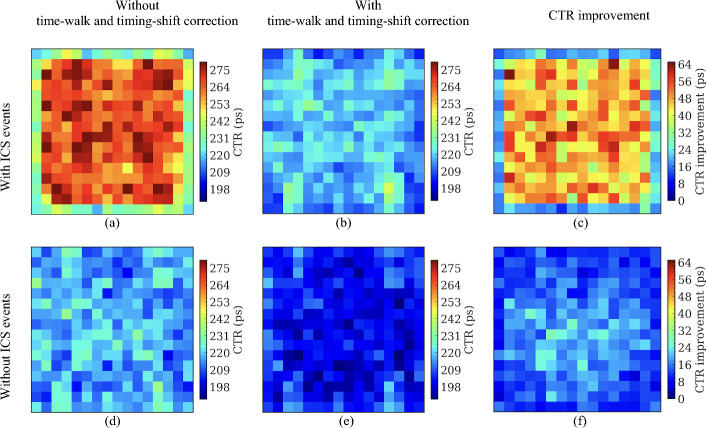
CTR for each of the selected 256 crystal pairs from detectors based on the 16 × 16 LYSO arrays with normal reflectors obtained (**a**, **d**) without and (**b**, **e**) with time-walk and timing-shift correction, and (**c**, **f**) the CTR improvement. CTR obtained (**a**, **b**) with ICS events was worse than CTR (**d**, **e**) without ICS events. A 400–650 keV energy window was applied to each crystal to select events

**Fig. 13:**
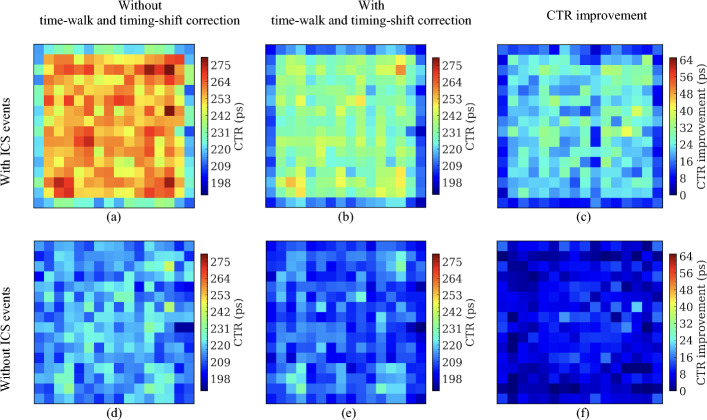
CTR for each of the selected 256 crystal pairs from detectors based on the 16 × 16 LYSO arrays with partial short reflectors obtained (**a**, **d**) without and (**b**, **e**) with time-walk and timing-shift correction, and (**c**, **f**) the CTR improvement. CTR obtained (**a**, **b**) with ICS events was worse than CTR (**d**, **e**) without ICS events. A 400–650 keV energy window was applied to each crystal to select events

Figures 11, 12, and 13 show the CTR of the selected crystal pairs for detectors based on the 8 × 8 LYSO arrays (Fig. 11), the 16 × 16 LYSO arrays with normal reflectors (Fig. 12), and the 16 × 16 LYSO arrays with partial short reflectors (Fig. 13), respectively. The average CTRs across all selected crystal pairs for the three types of detectors are listed in Table 2. It is evident that the CTRs were greatly improved by applying the time-walk and timing-shift corrections, especially for those with ICS events and the central crystals (Tables 2 and 3).

**Table 2:** The average CTRs of detectors based on the three types of LYSO arrays

CTR (ps)	8 × 8 LYSO array with a 3.2 mm pitch		16 × 16 LYSO array with a 1.6 mm pitch			
			Normal reflector		Partial short reflector	
	With ICS	Without ICS	With ICS	Without ICS	With ICS	Without ICS
Without correction	245 ± 11	207 ± 4	259 ± 16	217 ± 6	249 ± 16	216 ± 7
With correction	207 ± 5	197 ± 4	218 ± 7	202 ± 6	228 ± 11	209 ± 7
Improvement	38 ± 7	10 ± 2	41 ± 13	15 ± 5	21 ± 7	7 ± 4

**Table 3:** The average CTRs of the central 4 crystals in the detectors based on the 8 × 8 LYSO arrays and the central 16 crystals in the detectors based on the 16 × 16 LYSO arrays

CTR (ps)	8 × 8 LYSO array with a 3.2 mm pitch		16 × 16 LYSO array with a 1.6 mm pitch			
			Normal reflector		Partial short reflector	
	With ICS	Without ICS	With ICS	Without ICS	With ICS	Without ICS
Without correction	252 ± 3	208 ± 1	266 ± 7	220 ± 5	254 ± 6	218 ± 4
With correction	207 ± 2	196 ± 2	219 ± 5	200 ± 4	234 ± 6	208 ± 4
Improvement	45 ± 2	12 ± 2	47 ± 7	20 ± 3	20 ± 6	10 ± 4

By excluding the ICS events, the CTRs were also significantly improved. This occurred because, in the absence of ICS events, almost all scintillation light was detected by the SiPMs directly coupled to the crystal that detected the annihilation photons. Hence, the amplitude variation of SiPM signals was smaller compared to the case with ICS events, reducing the effect of time-walk.

The detectors based on the 8 × 8 LYSO arrays provided slightly better CTRs than detectors based on the 16 × 16 LYSO arrays. This was attributed to the larger size of the crystals, which allowed more scintillation light to reach the SiPMs (Fig. 9). However, the difference in average CTR values was relatively small, less than 20 ps. Compared to the detectors based on the 16 × 16 LYSO arrays with partial short reflectors, the detectors based on the 16 × 16 LYSO arrays with normal reflectors also provided slightly better CTR values, by less than 10 ps, due to less spread of the scintillation light among SiPMs.

An interesting phenomenon observed was that the CTRs of the edge crystals were superior to the center crystals, especially for the CTRs obtained with ICS events and without time-walk and timing-shift correction. We believe this is because: (1) fewer ICS events were assigned to the edge crystals, as events interacting with the edge crystals and escaping from the LYSO array were excluded by the energy window, and (2) events were primarily detected towards the front of the detectors due to the oblique angles of the annihilation photons at these locations and the use of the point source, resulting in less DOI effect. After excluding the ICS events, the CTRs for each crystal became more consistent.

### DOI resolution

**Fig. 14:**
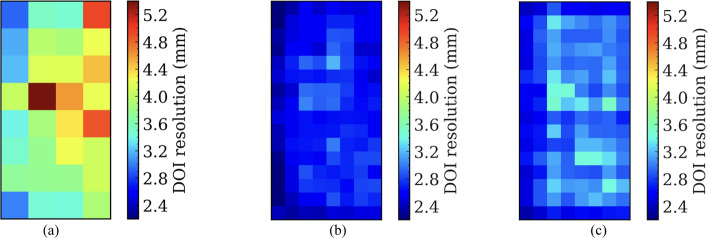
DOI resolution for each selected crystal of detectors based on (**a**) the 8 × 8 LYSO array, (**b**) the 16 × 16 LYSO array with normal reflectors, and (**c**) the 16 × 16 LYSO array with partial short reflectors

Figure 14 shows the DOI resolution for each of the selected crystals in the three types of detectors. Table 4 summarizes the average DOI resolution across all crystals and those in the first column.

**Table 4:** Average DOI resolutions of detectors based on the three types of LYSO arrays

DOI resolution (mm)	8 × 8 LYSO array with a 3.2 mm pitch	16 × 16 LYSO array with a 1.6 mm pitch	
		Normal reflector	Partial short reflector
First column crystals	3.4 ± 0.3	2.3 ± 0.1	2.5 ± 0.1
All selected crystals	3.9 ± 0.6	2.6 ± 0.2	2.9 ± 0.3

Due to the LYSO arrays being irradiated from the left side (Fig. 7) and the divergence of the collimating beam increasing with penetration depth, LYSO crystals closer to the ^22^Na source had better DOI resolutions. The crystals at the top and bottom rows in each LYSO array also had better DOI resolution due to fewer ICS events being assigned to those crystals.

Compared to detectors based on the 16 × 16 LYSO arrays, the detector based on the 8 × 8 LYSO array had worse DOI resolution due to the lower aspect ratio of the crystal elements. To obtain DOI information, some scintillation light was expected to be lost before reaching the SiPMs. The more the lost scintillation light, the better the DOI resolution. The higher the aspect ratio, the more likely scintillation light could be lost before reaching the SiPMs, either by being absorbed by the crystals, the reflectors, or the optical glue. Hence, for the detector based on the 16 × 16 LYSO array, more scintillation light was lost before reaching the SiPMs (Fig. 9), resulting in better DOI resolution.

Compared to the detector based on the 16 × 16 LYSO arrays with partial short reflectors, the detector based on the 16 × 16 LYSO arrays with normal reflectors had slightly better DOI resolution, possibly due to less scintillation light spread. However, the DOI resolution difference was insignificant, less than 10%.

## Discussion

In this study, we developed and compared the performance of dual-ended readout TOF-DOI PET detectors based on Hamamatsu S14161-3050-08 SiPM arrays and three types of LYSO arrays. The LYSO arrays had the same 20 mm thickness but different pitches (3.2 mm and 1.6 mm) and reflector arrangements (normal and partial short reflectors). PETsys TOFPET2 was used as the readout electronics. The performance of the detectors was compared in terms of flood histogram, energy resolution, CTR, and DOI resolution. All detectors showed excellent performance, making them promising candidates for developing next-generation whole-body PET and dedicated PET scanners, such as brain PET scanners, with uniform and high spatial resolution across the FOV.

The flood histograms show that all LYSO elements in all detectors were clearly resolved (Fig. 8). By adding a weighting factor to the conventional COG method, the crystal resolvability could also be improved, especially for the edge crystals of the detectors based on the 16 × 16 arrays. In our studies, we compared three values of the weighting factor (*n* = 1, 1.5, and 2), and the weighting factor *n* = 1.5 provided the optimal flood histogram for detectors based on the 16 × 16 arrays. The detector based on the 16 × 16 array with a 1.6 mm pitch and partial short reflectors (Fig. 8f) provided better crystal resolvability than the detector based on the 16 × 16 arrays with normal reflectors (Fig. 8e), making it more suitable for brain PET scanners that need high spatial resolution (Figs. 5b, c and 8e, f). However, the drawback of the 16 × 16 array with partial short reflectors is the complexity of the LYSO array fabrication, thereby increasing the cost slightly.

The energy resolution of 8.0 ± 0.6% for the detectors based on the 8 × 8 LYSO arrays (Fig. 10a) was better than that of the detectors based on the 16 × 16 LYSO arrays (11.4 ± 2.0% for normal reflectors (Fig. 10b) and 10.5 ± 2.0% for partial short reflectors (Fig. 10c)), due to the lower aspect ratio crystal elements of the 8 × 8 LYSO arrays, which was half of that of the 16 × 16 LYSO arrays. The detectors based on the 16 × 16 LYSO arrays had similar energy resolution. The energy resolution was not corrected for saturation due to the challenge and complexity of performing it for dual-ended readout detectors [49]. However, considering that dual-ended readout detectors were used, we believe that the saturation was less than those obtained from single-ended readout detectors, and the energy resolution will not change significantly if saturation is corrected.

Because the lower aspect ratio of the 8 × 8 LYSO arrays improves the amount of scintillation light reaching the SiPMs, detectors based on this type of LYSO array had better CTR than detectors based on the 16 × 16 LYSO arrays (Tables 2 and 3). The detectors based on the 16 × 16 LYSO arrays with normal reflectors had better CTR than those based on the 16 × 16 LYSO arrays with partial short reflectors due to less scintillation light sharing among SiPMs. The application of time-walk and timing-shift corrections significantly enhanced CTR. The CTRs improved by 38 ps and 41 ps for the detectors based on the 8 × 8 LYSO arrays and 16 × 16 LYSO arrays (Tables 2 and 3), respectively, indicating that time-walk and timing-shift corrections are essential for dual-ended TOF-DOI readout detectors. Excluding the ICS events also improved the CTR (Tables 2 and 3). However, 48.4%, 65.3%, and 58.5% of coincidence events were excluded from the detectors based on the 8 × 8 LYSO arrays, the 16 × 16 LYSO arrays with normal reflectors, and the 16 × 16 LYSO arrays with partial short reflectors, respectively, but the CTR improved by only ~ 20 ps when the time-walk and timing-shift corrections were performed (Tables 2 and 3).

The circular mask-based method was used in our studies to remove ICS events [47]. The drawback of using this method is that the ICS event ratio was underestimated in our studies, especially for the detectors based on the 16 × 16 LYSO arrays, due to the crystal spots not having a circular shape, particularly for detectors based on the 16 × 16 LYSO arrays with partial short reflectors (Fig. 5). To more accurately identify ICS events, it is crucial to develop more sophisticated algorithms, such as deep learning-based methods [47, 50]. However, the image quality of the PET scanner is determined by both its sensitivity and CTR. Although excluding ICS events can improve the CTR and the spatial resolution, the sensitivity of the scanners will be significantly decreased. A better approach is to identify and keep ICS events and model them properly during image reconstruction.

The DOI resolution across the selected crystals of the detectors based on the 8 × 8 LYSO array, the 16 × 16 LYSO array with normal reflectors, and the 16 × 16 LYSO array with partial short reflectors were 3.9 ± 0.6 mm, 2.6 ± 0.2 mm, and 2.9 ± 0.3 mm (Table 4), respectively, which are quite good for whole-body PET and brain PET applications. The DOI resolutions were not as good as our previous studies, based on the same crystal thickness but smaller pitches [26], due to the lower aspect ratio of the LYSO elements used in this study and the different beam width. We are currently investigating the relationship between the DOI resolution and the aspect ratio using LYSO arrays with different pitches and thicknesses through experiments and simulations. The results will be reported in the near future.

The commercially available PETsys TOFPET2 electronics was used in our studies, which, while scalable for use at a system level, cannot provide a CTR as high as other studies that were based on single scintillation crystals and radio frequency (RF) readout [51–53]. Other reasons that the CTR in our studies was not as good as single-crystal-based results are that 1) in single-crystal-based studies, the bias voltage and threshold used for timing pick-off can be carefully optimized to achieve the best CTR [54, 55]. However, in our studies, due to the non-uniform breakdown voltage of the SiPMs, the slight difference of LYSO elements in the same array, and the performance difference among the channels of the readout electronics, it was not possible for us to optimize the bias voltage and the timing pick-off threshold for each SiPM and each readout channel, and 2) in single-crystal-based studies, there are no ICS events. We believe that the upcoming release of the improved PETsys TOFPET3 will lead to improvements in CTR [56], and we will repeat our measurements when it becomes available.

Overall, the detectors based on the 8 × 8 LYSO arrays with a 3.2 mm pitch had better energy resolution and CTR but worse spatial resolution and DOI resolution than the detectors based on the 16 × 16 LYSO arrays. The detectors based on the 16 × 16 LYSO arrays with normal reflectors had better energy resolution, CTR, and DOI resolution but slightly worse spatial resolution than those based on the 16 × 16 LYSO arrays with partial short reflectors.

## Conclusion

Three types of TOF-DOI PET detectors based on the dual-ended readout method and LYSO arrays with two different pitches (3.2 mm and 1.6 mm) and two different reflector configurations (normal reflectors and partial short reflectors) were evaluated and compared for whole-body PET and dedicated PET scanners, such as brain PET scanners. All detectors showed excellent performance, and all LYSO elements in the detectors could be clearly resolved. The detectors based on the 8 × 8 LYSO arrays with a 3.2 mm pitch provided the best CTR of 207 ± 5 ps, making it ideal for whole-body PET scanners. The detectors based on the 16 × 16 arrays with a 1.6 mm pitch and normal reflectors achieved better CTR (218 ± 7 ps) and DOI resolution (2.6 ± 0.2 mm) than the detectors based on the 16 × 16 LYSO arrays with partial short reflectors (228 ± 11 ps for CTR and 2.9 ± 0.3 mm for DOI resolution) but had slightly worse crystal identification ability. Hence, both types of detectors based on the 16 × 16 LYSO arrays with a 1.6 mm pitch are suitable for high-resolution PET applications such as brain and small-animal PET scanners and can also be used for whole-body PET scanners with uniform and high spatial resolution across the FOV. Excluding ICS events could improve the CTR, but the improvement was relatively marginal (less than 10% or 20 ps). In comparison, ~ 50% and ~ 60% of coincidence events were excluded from detectors based on the 8 × 8 LYSO arrays and 16 × 16 LYSO arrays, respectively, which will significantly reduce the sensitivity of the PET scanners. Hence, more research needs to be performed to investigate the effect of excluding the ICS events at a system level when these detectors are used to develop PET systems.

## Data Availability

The datasets used and/or analyzed during the current study are available from the corresponding author on reasonable request.

## Abbreviations

ADC: Analog-to-digital converter
ASIC: Application-specific integrated circuit
BaSO₄: Barium sulfate
CTR: Coincidence timing resolution
COG: Center-of-gravity
DOI: Depth-of-interaction
FOV: Field-of-view
FWHM: Full width at half maximum
ICS: Inter-crystal scatter
LED: Leading-edge discriminator
LOR: Line-of-response
LSO: Lutetium oxyorthosilicate
LYSO: Lutetium-yttrium oxyorthosilicate
PET: Positron emission tomography
P.P.: Photopeak position
RF: Radio frequency
SiPM: Silicon photomultiplier
SNR: Signal-to-noise ratio
TOF: Time-of-flight
